# Modulation of contractions in the small intestine indicate desynchronization via supercritical Andronov–Hopf bifurcation

**DOI:** 10.1038/s41598-020-71999-4

**Published:** 2020-09-15

**Authors:** Sean P. Parsons, Jan D. Huizinga

**Affiliations:** Farncombe Family Digestive Health Research Institute, McMaster University, 1280 Main Street West, HSC 3N5, Hamilton, ON L8S 4K1 Canada

**Keywords:** Nonlinear dynamics, Oscillators, Biological physics, Nonlinear phenomena

## Abstract

The small intestine is covered by a network of coupled oscillators, the interstitial cells of Cajal (ICC). These oscillators synchronize to generate rhythmic phase waves of contraction. At points of low coupling, oscillations desynchronise, frequency steps occur and every few waves terminates as a dislocation. The amplitude of contractions is modulated at frequency steps. The phase difference between contractions at a frequency step and a proximal reference point increased slowly at first and then, just at the dislocation, increased rapidly. Simultaneous frequency and amplitude modulation (AM/FM) results in a Fourier frequency spectrum with a lower sideband, a so called Lashinsky spectrum, and this was also seen in the small intestine. A model of the small intestine consisting of a chain of coupled Van der Pol oscillators, also demonstrated simultaneous AM/FM at frequency steps along with a Lashinsky spectrum. Simultaneous AM/FM, together with a Lashinsky spectrum, are predicted to occur when periodically-forced or mutually-coupled oscillators desynchronise via a supercritical Andronov–Hopf bifurcation and have been observed before in other physical systems of forced or coupled oscillators in plasma physics and electrical engineering. Thus motility patterns in the intestine can be understood from the viewpoint of very general dynamical principles.

## Introduction

**Figure 1:**
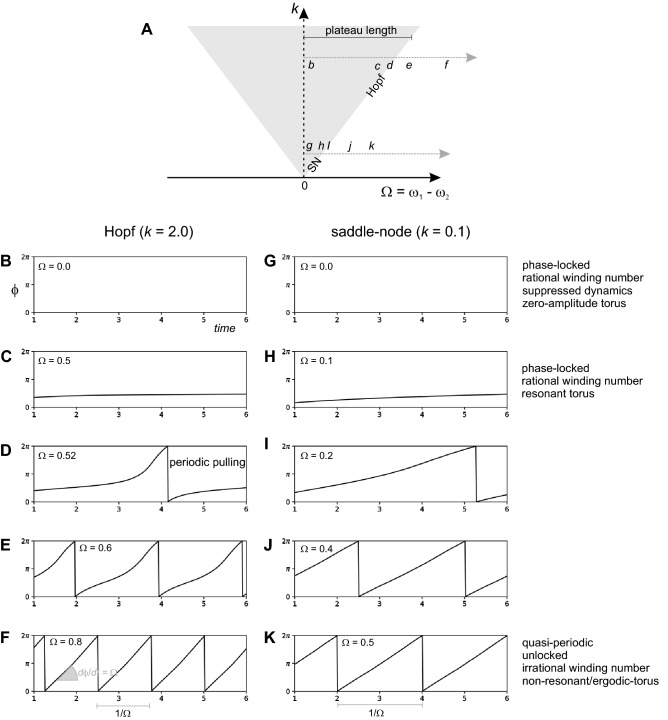
Synchronization. (**A**), Phase-locking graph for two oscillators. The y-axis (*k*) is the coupling strength and the x-axis (Ω) is the detuning, the difference between the natural frequencies of the oscillators (ω_1_ and ω_2_). In the literature the x-axis is sometimes plotted as ω_1_/ω_2_ and uni-directional coupling is analysed, with oscillator #1 as the force/driver and oscillator #2 as the forced/driven. Synchronization occurs within a triangular shaped region (grey shading) of the phase-locking graph: the Arnold tongue. Proceeding distally along an intestine frequency plateau (horizontal black line) Ω starts at 0 and increases until the border of the Arnold tongue is reached when a new plateau forms (a frequency step occurs). Therefore plateau length is proportional to the width of the tongue and increases with *k*. Synchronization equates to "phase-locking": a fixed phase difference (ϕ) between the oscillators over time, *d*ϕ/*dt* = 0. At the centre of the tongue (Ω = 0), ϕ = 0. As one moves further from the centre ϕ increases. At the edge of the tongue phase becomes non-constant (*d*ϕ/*dt* = non-zero) and far from the tongue *d*ϕ/*dt* = Ω. At the edge of the tongue, the behaviour of ϕ(*t*) depends on the value of *k*. This is illustrated by two transects of the tongue *b–f* and *g–k* (dashed vertical lines). (**B–F**) and (**G–K**) show ϕ(*t*) for their respective positions on the transects, with ϕ plotted as its modulus with respect to 2π (a single cycle). (**B–F**) shows desynchronization proceeding through a supercritical Andronov-Hopf bifurcation (high *k*). At the border of the tongue ϕ(*t*) is very non-linear (*d*ϕ/*dt* = non-constant): this is called "periodic pulling" or "frequency modulation" (*d*ϕ/*dt* = the instantaneous frequency of the forced oscillator). (**G–K**) shows desynchronization proceeding through a saddle-node (SN) bifurcation. At the border of the tongue ϕ(*t*) remains almost linear (*d*ϕ/*dt* ~ constant). All trajectories were solved numerically from the truncated equation for ϕ: *d*ϕ/*dt* = Ω—*k*((ω_2_^2^/ω_1_) sin ϕ)^19^.

Oscillators occur throughout nature, from circadian clocks to metabolic pathways to fireflies to superconductors. Overlying this diversity is a universal phenomenon: synchronization^1–3^. In isolation an oscillator oscillates at its "natural" frequency (ω_0_). Coupled together, oscillators of differing ω_0_ have a tendency to oscillate together at the same frequency: this is synchronization. Synchronisation is primarily dependent on two factors: (i) The difference between the ω_0_ of the oscillators, called the detuning (Ω) and; (ii) the strength of the coupling between oscillators (*k*). With no coupling (*k* = 0) synchronization only occurs (by default) when Ω = 0. As coupling increases the range of detuning over which synchronization occurs increases. This relationship is commonly plotted on a graph of *k* against Ω (Fig. 1A). The region of synchronisation on this "phase-locking graph" is an inverted triangle with its apex at (Ω = 0, *k* = 0). This triangle is called the Arnold tongue^1^ after Vladimir Arnold.

Two oscillators can be coupled bi- or uni- directionally. In bi-directional coupling the synchronised frequency (ω_s_) tends toward the higher ω_0_ and the oscillator with the lower ω_0_ is said to be "pulled" toward this frequency. In uni-directional coupling one oscillator forces or "drives" the other and as the driving frequency is fixed, ω_s_ can only be the ω_0_ of the driver. *k* is equivalent to the amplitude (force) of the driver. Uni-directional coupling is a common in many electrical devices and physical experiments, with the phase-locking graph presented as force vs driving frequency. A population of synchronized oscillators can be treated as a single "lumped" oscillator with a ω_0_ = ω_s_. This is pertinent when we talk about or model coupled oscillators on a macroscopic scale, when we know that physically those oscillators are discrete at a microscopic scale, such as biological cells.

A corollary of synchronization is that the phase difference (ϕ) between oscillators is constant: *d*ϕ/*dt* = 0. The oscillators are said to be "phase-locked". Phase-locking has the consequence that oscillations appear to travel between oscillators as rhythmic waves, "phase waves"^4,5^. The phase wave velocity = *d *× ω_s_/ϕ, where *d* is the distance between oscillators. ϕ increases (velocity decreases) in proportion to Ω. Phase waves occur in many oscillatory chemical systems: the iron-wire/nitric acid, malonic acid and Belousov–Zhabotinsky reactions^6^. Outside the Arnold tongue ϕ increases linearly as the oscillators run at their own ω_0_: by definition *d*ϕ/*dt* = constant = Ω. This is called "quasi-periodicity" (rather confusingly as both oscillators are perfectly periodic). *d*ϕ/*dt* is the difference in the "instantaneous" frequencies of the oscillators.

The universality of synchronisation in nature is reflected in its mathematics. This math has its roots in Huygens' analysis of pendulums in the seventeenth century; matured during the early twentieth century in response to the demands of the burgeoning field of radio-communication; and then found its most universal expression in the theory of nonlinear dynamics^7^. From this vantage point one can make very general statements about a driven oscillator's behaviour near the border of the Arnold tongue^8^. Two different things can happen:

i. At high *k* the oscillator goes through a supercritical Andronov–Hopf bifurcation (Fig. 1B–F). This means:
  - The amplitude of the oscillation repeatedly waxes and wanes, or "beats" at a frequency that approaches Ω. This is called amplitude modulation (AM). The bounds of the modulated oscillations, are called its "envelope".
  - ϕ changes nonlinearly, *d*ϕ/*dt* is non-constant. The oscillator's instantaneous frequency is pulled back and forth between ω_s_ (*d*ϕ/*dt* = 0) and ω_0_ (*d*ϕ/*dt* = Ω), periodically at the beat frequency. This is called "periodic pulling" or "frequency modulation" (FM).
ii. At low *k* the oscillator goes through a saddle-node bifurcation (Fig. 1G–K). This means:
  - There is no AM.
  - ϕ changes linearly, *d*ϕ/*dt* is constant. There is no FM.

Apart from measuring ϕ and amplitude, the two scenarios can be distinguished by the oscillator's Fourier frequency spectrum^9^. In the saddle-node bifurcation (ii) there is a single frequency peak that shifts from ω_s_ to ω_0_ as one proceeds across the border of the tongue. In the supercritical Andronov–Hopf bifurcation (i), periodic pulling of the instantaneous frequency broadens the spectra: a number of decaying "sideband" peaks form to one side of ω_s_, at intervals of Ω,

1 $$\omega_{peak} = \omega_{s} + n\Omega .$$

If ω_s_ > ω_0_ the sideband is to the left of ω_s_ (n < 0; "lower-sideband"); if ω_s_ < ω_0_ the sideband is to the right (n > 0; "upper-sideband"). The other sideband is said to be "suppressed". The single sideband spectrum is a mathematical consequence of simultaneous AM-FM^10–13^ and is known as a Lashinsky spectrum in plasma physics after Herbert Lashinsky^14^. Such spectra have been observed in a wide range of driven oscillators: gallium-arsenide microwave generators^15^; tunnel diodes^16^; plasma Q-machines^10,11^; magnetrons^17^; neon bulbs^18^; filament cathodes^19^; Rijke tube acoustic devices^20^. It is an engineered quality in many communication devices^21^.

Waves of contraction travel down the small intestine, pushing content from the stomach toward the colon (in a "distal" direction). These waves are phase waves and the oscillators are the interstitial cells of Cajal of the myenteric plexus (ICC-MP)^22^. The ICC-MP form a one-cell thick network that covers the length and circumference of the small intestine^23^. The membrane potential of each ICC-MP oscillates and these oscillations ("slow waves") depolarize the muscle causing it to contract. The ICC-MP are coupled by gap junctions, ionic pores between the cytoplasm of adjacent cells.

The distal direction of the intestine's phase waves is due to a downward gradient of ω_0_. The gradient also means that the phase waves are pulled into a series of "plateaux" of constant frequency, separated by abrupt "frequency steps"^24,25^. Proceeding distally across a plateau, the detuning between ω_s_ (the plateau's lumped ω_0_) and the ω_0_ of the point distal to it, increases from zero until synchronization fails, resulting in a frequency step. ω_0_ at the step then becomes the ω_s_ of the next plateau. This corresponds to moving from the centre of the Arnold tongue, right, until one crosses its border (Fig. 1). If *k* is greater, the Arnold tongue is wider and so the plateau is longer. We recently demonstrated this experimentally: reducing *k* with the gap junction blocker carbenoxolone, reduces plateau size^24^. Also a step is induced by a local decrease in *k*, a vertical movement down the phase-locking diagram, cutting across the tongue border^25^. Again this has been demonstrated experimentally by local "pinch" decoupling^26^.

Most phase waves "travel" the whole length of the intestine, but at the frequency step every ω_s_/Ωth wave terminates—i.e. a wave terminates every 1/Ω seconds (2π/Ω with angular frequencies in radians/s)^24^. This termination is called a "dislocation" of the wave train. Each dislocation is associated with a comet-shaped wave of increased contraction interval^26^, i.e. frequency modulation, and it is these quantal "interval waves" that merge to form the frequency plateau. Also contraction amplitude is modulated at the frequency step—it waxes and wanes. Here we show that amplitude and frequency modulation at the frequency step has the signature of a supercritical Andronov–Hopf desynchronisation.

## Results

### The small intestine

All experiments were carried out in the presence of 0.5 mM lidocaine, which blocks the activity of neurons in the intestine. Under these conditions every depolarisation wave of the ICC is followed by a contraction of the muscle. Contraction amplitude is a function of the probability of all-or-none action potential firing in individual muscle cells, and this in turn is a function of the amplitude of the ICC generated slow wave. Thus slow wave and contraction amplitude will correlate.

**Figure 2:**
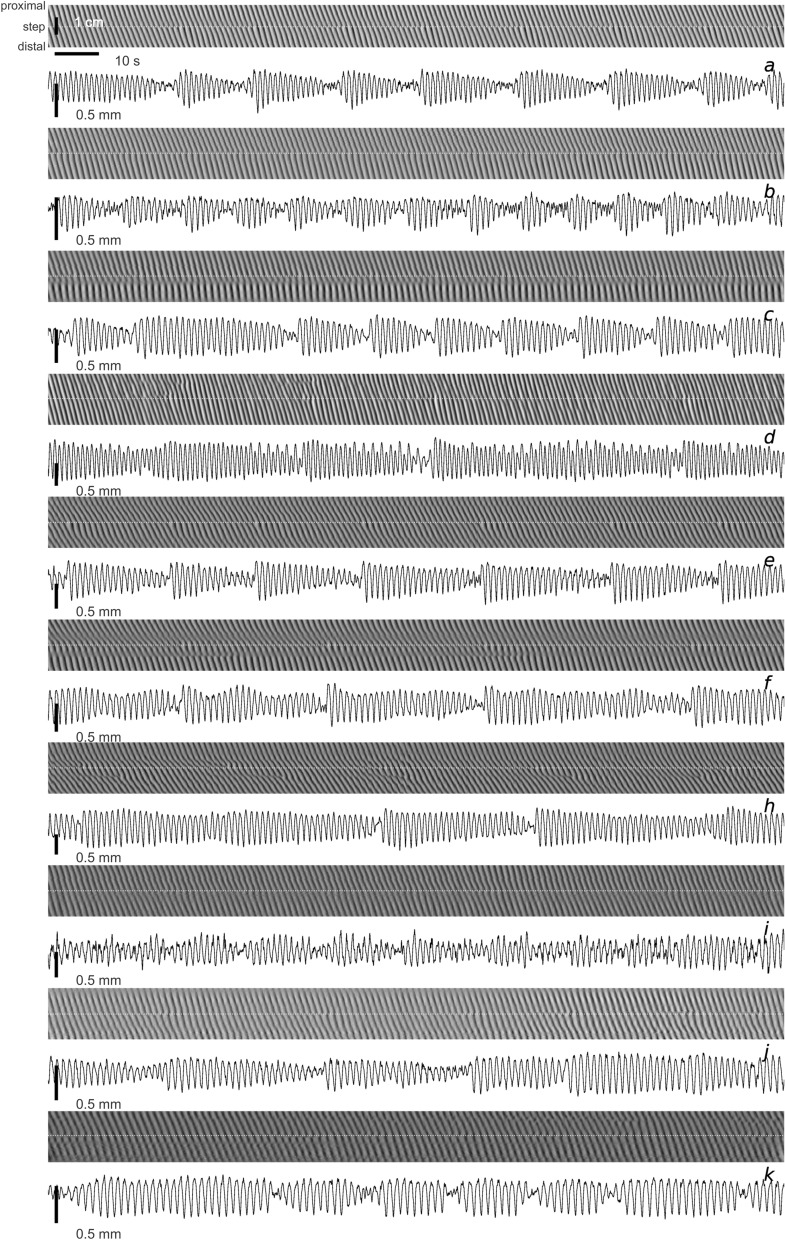
Amplitude modulation at frequency steps. Ten examples, from different intestines, of paired diameter maps (upper panels) and diameter profiles (lower panels) taken along the white dotted line of those maps. All DMaps are 3 cm by 3 min. Scales where not indicated are the same as in the first panel. The frequency step is marked by the repeated occurrence of terminating waves (dislocations). At the step, diameter waxes and wanes: amplitude modulation. The envelope of the modulation reaches a minimum at each dislocation. Most often the envelope then increases (waxes) very fast before gradually decreasing (waning) to the next dislocation.

Contraction amplitude, measured as a reduction in intestinal diameter, was modulated at frequency steps (Fig. 2)^24^. Most commonly amplitude modulation was time asymmetric, with the waxing phase being much faster, often almost instantaneous, so that the amplitude envelope had the shape of an arrowhead pointing forward in time. The waning phase, was usually slowest at the beginning and then sped up towards the dislocation. Apart from this, modulation varied considerably both between intestines and between steps in the same intestine. The envelope minima were always associated with wave dislocations. Thus the beat frequency was equal to the dislocation frequency.

**Figure 3:**
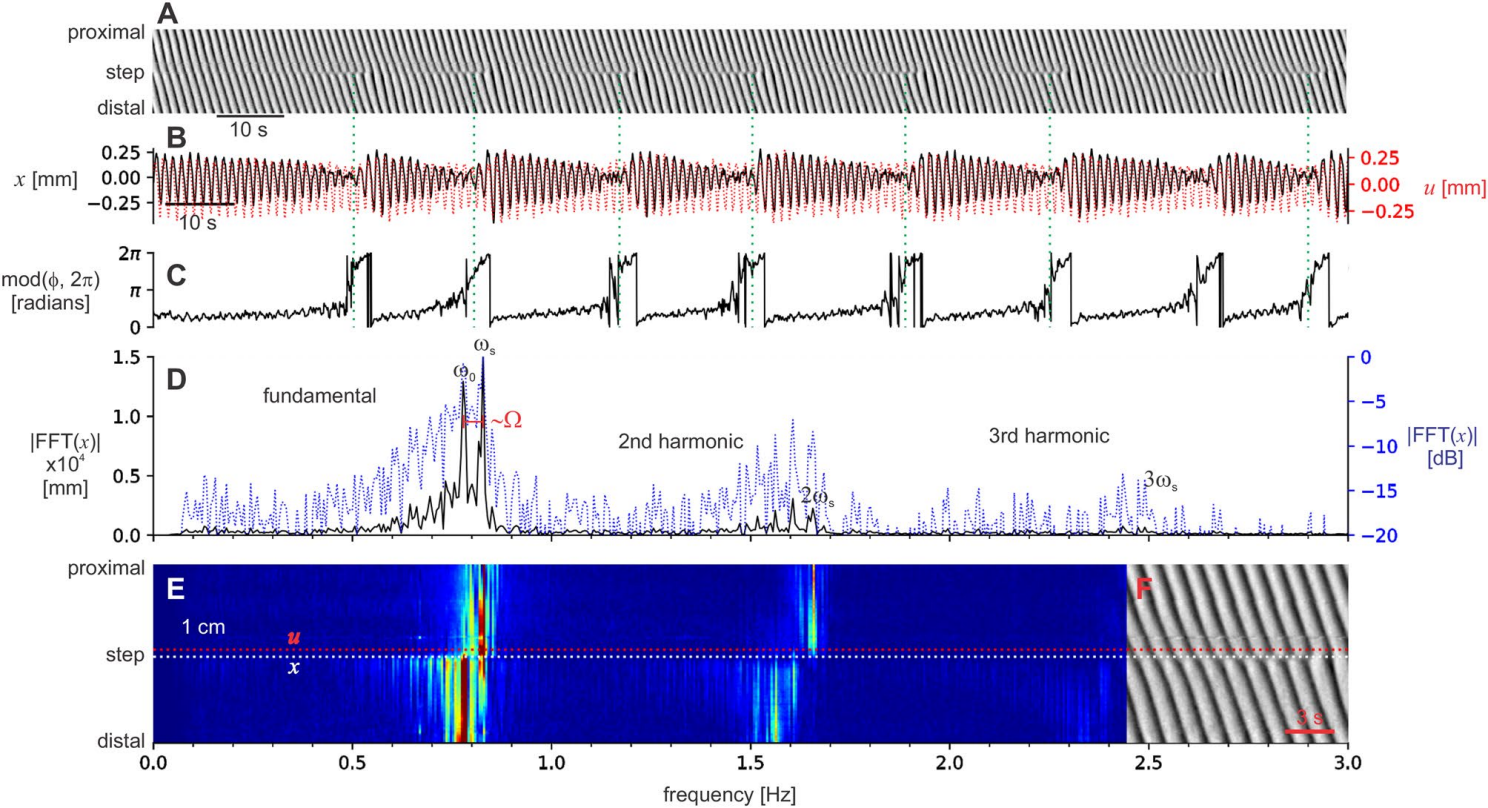
Signal analysis of the intestine. (**A**) The same diameter map at the top of Fig. 1 (intestine *a*). (**B**), diameter at the step (*x*, black line) and 1.2 mm proximal to that point (*u*, red line). Due to bandpass filtering (“Methods”) the signals oscillate about zero, a level that corresponds to the mean diameter in the unfiltered signal. Green dotted lines indicate the times of dislocations in the diameter map. (**C**) The phase difference between *x* and *u* (ϕ) rapidly increases at the end of each beat. (**D**) Fourier spectrum of *x* on an absolute scale (black line) and a logarithmic scale (dB, blue line). The frequency axis is below (**E**). To the left of the proximal plateau frequency (ω_s_) peak is a lower-sideband of decaying peaks at intervals of ~ Ω. The upper-sideband is suppressed. This pattern is repeated for at least the second harmonic. The cut-offs of the bandpass were 0.1 and 5 Hz, well outside the range of the fundamental and harmonics. (**E**) Fourier spectra across the step (colored absolute scale). The lines for *x* and *u* are indicated and at the right (**F**) is a section of the corresponding diameter map. Proximal to the step the dominant frequency is ω_s_. Distal to the step the lower-sideband decays with distance as the ω_0_ at the step became the ω_s_ of the distal plateau.

We measured the diameter at the step (*x*) and just proximal to it (*u*) (Fig. 3A,B). *u* is the plateau oscillator with the frequency ω_s_, and *x* is the oscillator that is loosing synchrony with the plateau, being pulled toward its ω_0_. The phase difference between *x* and *u* (ϕ) showed periodic pulling (Fig. 3C). ϕ increased very slowly over most of the beat but at the point of the dislocation, just as the beat had reached a minimum, it then rapidly increased. The Fourier spectra of *x* showed the lower-sideband structure expected for ω_s_ > ω_0_ (Fig. 3D). The *n* = 0 (ω) and *n* = − 1 (~ ω_0_) peaks were clearly distinguished. The lower frequencies (n < − 1) merged together, but were clearly decaying. Intestinal contraction waves are not quite harmonic (like a sine wave) and so would be expected to have harmonics, peaks at multiples of their fundamental frequency. This was the case: there were second and third harmonics. The second harmonic was just large enough to distinguish the lower-sideband.

**Figure 4:**
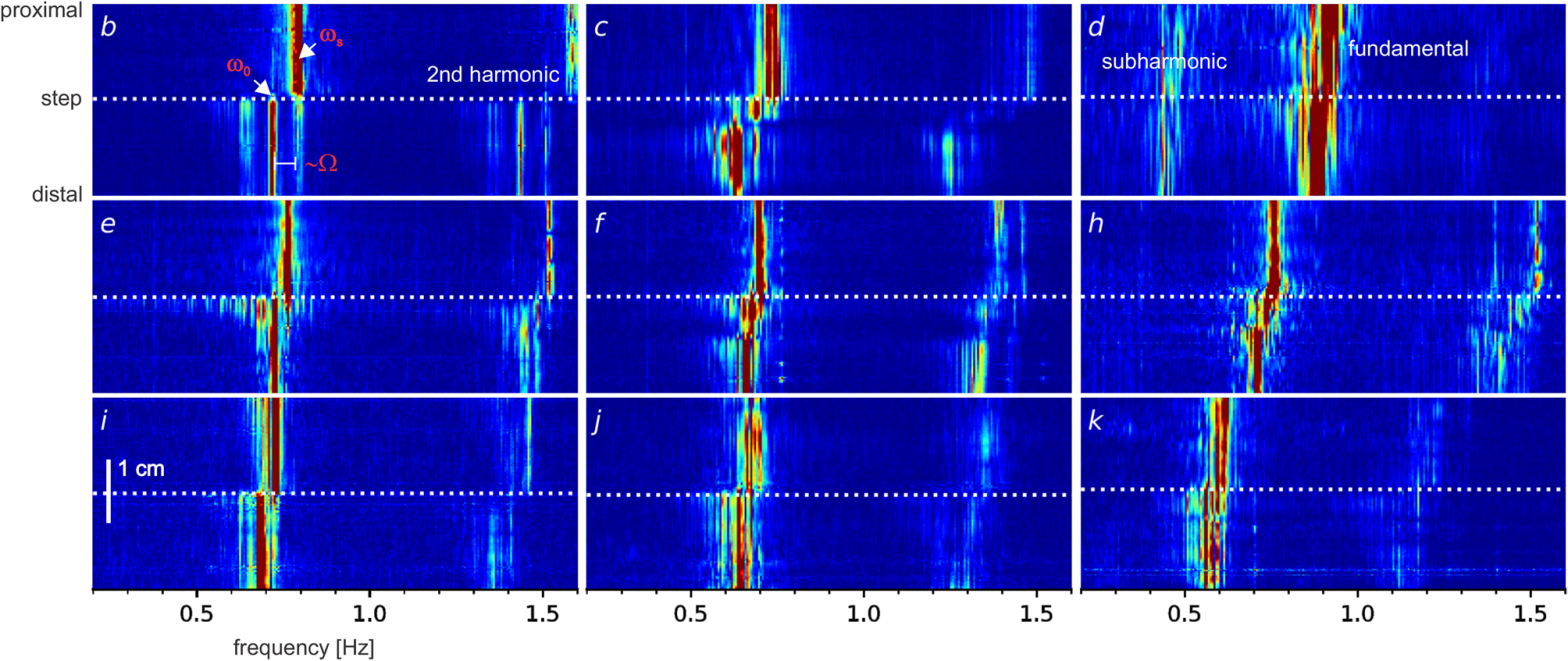
Fourier spectra of intestine frequency steps. Fourier spectra for each of the lowest nine DMaps shown in Fig. 2 (corresponding letters, *b* to *k*). Colored absolute scale. White dotted lines indicate the position of the diameter traces in Fig. 2, the step position. The proximal plateau frequency is ω_s_. At the step, to the left of ω_s_ is a lower-sideband of decaying peaks at intervals of ~ Ω. Distal to the step the lower-sideband decays with distance as the ω_0_ of the step became the ω_s_ of the distal plateau. Notice the presence of a subharmonic in *d*.

When the Fourier spectra for the region immediately about the step (1.5 cm either side) was calculated, it became clear that the lower-sideband extended some distance from the step (Fig. 3E). Proximal to the step the plateau frequency (ω_s_) stood clear. At the step, the lower-sideband appeared immediately. It was most extended at the step and then decayed away as the ω_0_ of the step became the ω_s_ of the new plateau. The positions of individual peaks did not change with distance from the step (detuning) as would occur in a saddle-node bifurcation. This pattern was apparent in the spectra of all the example steps shown in Fig. 2 (Fig. 4).

### Van der Pol model

The Van der Pol (VdP) is a common simple model of nonlinear oscillators^22^. Since its creation in 1926^27^ thousands of papers and books have analysed its dynamics in various configurations, including coupled and driven, and used it to model oscillatory phenomena as varied as fluid turbulence^28^ and plasmas^29^. Robert Fitzhugh showed that the VdP is a simplified ("reduced") version of Hodgkin and Huxley's model of the giant squid axon, the progenitor of all modern models of cellular electrophysiology^30^. To model the oscillators of the small intestine we coupled 200 VdP oscillators into a chain (“Methods”). They were coupled through the equation for their membrane potential variable in a manner equivalent to resistive gap-junction coupling^22^.

**Figure 5:**
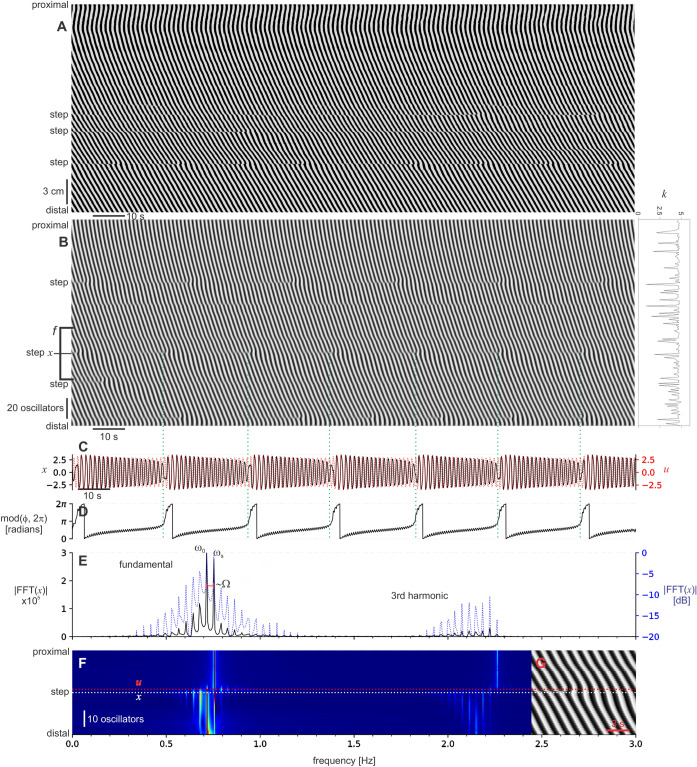
The coupled VdP model. (**A**) a diameter map of a small intestine, contrast adjusted to highlight the wave pattern. (**B**) Spatial–temporal map of "membrane potential" (*v*) of the coupled VdP model. The graph at the right shows coupling strength (*k*) along the chain. (**C**) Membrane potential at the point marked *x* in (**B**) (black line) and two oscillators proximal to this point (*u*, red line). Green dotted lines indicate the times of dislocations in the map. 9**D**) The phase difference between *x* and *u* (ϕ) rapidly increases at the end of each beat. (**E**) Fourier spectrum of *x* on an absolute scale (black line) and logarithmic scale (dB, blue line). The frequency axis is below (**F**). To the left of the proximal plateau frequency (ω_s_) is a lower-sideband of decaying peaks at intervals of ~ Ω. The upper-sideband is suppressed. This pattern is repeated for at the third harmonic. (**F**) Fourier spectra across the step (colored absolute scale). The lines for *x* and *u* are indicated and at the right (**F**) is a section of the corresponding diameter map. Proximal to the step the dominant frequency is ω_s_. Distal to the step the lower-sideband decays with distance as the ω_0_ at the step became the ω_s_ of the distal plateau.

The coupled VdP produced a pattern of phase waves very similar to the small intestine (Fig. 5A,B). Phase waves traveled from the proximal to the distal end of the chain, down a linear gradient in ω_0_ (0.9 to 0.75 Hz; similar to values in the small intestine^24^). As in our previous model of chained phase oscillators^25^ coupling strength (*k*) was varied along the length of the chain, so that steps with their attendant dislocations, occurred at fixed positions of low *k*.

When the model was analysed in the same manner as the intestine (Fig. 3), we find the same pattern (Fig. 5C–F). Amplitude waxed fast following a dislocation and then gradually waned to the next dislocation (Fig. 5C). The frequency modulation was remarkably similar to the intestine. Over most of the beat ϕ increased only very slowly, then just at the point of the dislocation, as the envelope reached its minimum, it increased rapidly (Fig. 5D). The Fourier spectra was also similar to the intestine. The peak at ω_s_ had a decaying lower-sideband, with peaks at intervals of Ω (Fig. 5E). As this was a model the peaks were much better separated then the intestine. There was a third harmonic but no second. When the Fourier spectrum was calculated around the step, the decay of the lower-sideband away from the step was similar to the intestine (Fig. 5F). It was most extended at the step and then decayed away as the ω_0_ of the step became the ω_s_ of the new plateau. The positions of individual peaks did not change with distance from the step (detuning).

## Discussion

Amplitude and frequency modulation and the Lashinsky spectrum are signatures of desynchronisation via the supercritical Andronov–Hopf bifurcation. Here we have shown that they are present at frequency steps in the small intestine. They also occur at frequency steps in a simple coupled oscillator model of the intestine.

Diamant and Bortoff suggested that AM at the frequency step resulted from the superposition (adding up) of oscillations of differing frequency either side of the frequency step^31^. When two sine waves of different frequency (ω_1_ and ω_2_) are added up, the result is a sine wave with their mean frequency, the amplitude of which waxes and wanes at the difference in frequency (ω_1_–ω_2_). Each wax-wane cycle is a "beat" and ω_1_–ω_2_ is the beat frequency^4^. The beat frequency is audible as a "difference tone" in a musical instrument, and in this guise AM by superposition has been recognised for centuries^32,33^. But we were suspicious of this explanation for AM in the small intestine for two reasons:

1. Superposition of unlocked oscillations (i.e. two oscillations at constant but differing frequencies) gives time-symmetric modulation: the wax envelope looks like a mirror image of the wane^4^. In the intestine the wane is gradual whereas the wax is sudden (Figs. 3, 4).
2. Mathematical modeling with chains of oscillators reproduced waxing-waning (Fig. 5), but such models do not include superposition: there is no adding-up of the output (state) variables of different oscillators.

In agreement with our data, Diamant and Bortoff observed mostly time-asymmetric AM in their electrical (i.e. slow wave) recordings of the cat small intestine, with a rapid wax and slow wane^31^. Just like us they modeled the intestine with a chain of coupled VdP but did not see AM, despite the presence of frequency plateaux^34^. Instead they added together the amplitudes of two neighboring VdP and showed an asymmetrically modulated amplitude. Note this does not negate reason (ii), above, as the summated oscillators were not unlocked. Such chain models of the intestine, consisting of coupled VdP, Fitzhugh–Nagumo, or Hodgkin–Huxley oscillators, became very popular during the 1970s^22^ but no one else appears to have looked at AM in these models.

Suzuki and colleagues carried out an experimental investigation of electrical (slow wave) AM in the small intestine of cats and rabbits^35^. The amount of AM asymmetry varied: some beats ("spindles") waxed fast and waned slow, but some appeared more symmetric. They presented Fourier spectra of two example traces (Fig. 5). The second of these appears to have an upper-sideband, but the frequency resolution of the spectra is low so it is not entirely straight forward to interpret. They supported Diamant and Bortoff's theory of superposition.

We published an experimental study of slow wave AM in the mouse small intestine^36,37^. The beats appeared mostly symmetric. Wavelet, rather then Fourier, spectra were calculated^36,37^ and these would not be expected to show side-bands due to the temporal localisation of the wavelet: the beat frequency is represented as a peak at that frequency, rather than a sideband of the oscillation frequency. Beats were induced by short-chain fatty acids and as these had been previously shown to induce a segmentation motor pattern^38^ it was suggested that these beats were associated with segmentation. Also we suggested a mechanism for the AM: phase-amplitude coupling^37^. We suggested the phase of a low (beat) frequency oscillator, hypothesised to be the ICC of the deep muscular plexus, modulated the amplitude of the ICC-MP. It may be that AM induced by short-chain fatty acids or associated with segmentation is a different phenomenon from AM at frequency steps: certainly the symmetric envelopes of the former might suggest this. If not, then in a sense "phase-amplitude coupling" is the correct name: amplitude (AM) is coupled to ϕ (FM) in the truncated equations of a driven oscillator (see below).

We have already recognised FM at the frequency step, in the form of interval waves^26^. When we measured the intervals between individual contraction waves we found a comet-shaped region of increased interval "propagated" distally from every dislocation. This is equivalent to the nonlinear modulation of ϕ, the change in instantaneous frequency at the step. ϕ changes rapidly right after the dislocation, in both the intestine (Fig. 3) and the coupled VdP (Fig. 5), and this matches with the locus of the interval wave. The frequency plateau is not a monolithic spatial–temporal entity, but rather made up of the quantal interval waves whose expanding tails decay and merge together. This corresponds to the contraction of the lower-sideband away from the step, with eventually only ω_0_, the ω_s_ of the new plateau, remaining (Figs. 3, 4).

The mathematical analysis of coupled oscillators is fiendishly difficult, particularly if those oscillators are nonlinear. Mathematicians have to make approximations to get anywhere. One approximation is to ignore amplitude, to assume that the amplitudes of the oscillators do not change when coupled. This is called the "weakly coupled" approximation, because the stronger coupling is, the more likely it will effect oscillation amplitude^39^. "Weakly coupled oscillator" models, where the only variable is the phase of the oscillators were developed by Winfree and Kuramoto in the 1970s and 1980s^39^ and were used by us to model the small intestine^25,26^. But they are obviously of no use in modeling amplitude modulation.

Another good approximation can be made for forced oscillators (uni-directional coupling)^4,40^:

2 $$x_{2} (t) = A(t)\sin (\omega_{1} t - \phi (t))$$

*x*_2_(*t*) is the amplitude of the driven oscillator. *A*(*t*) is the oscillator's amplitude envelope, i.e. its amplitude modulation. ϕ(*t*) is the phase difference between the oscillator and its driver, i.e. the quantity we have been measuring in the intestine and that amounts to frequency modulation. ω_1_ is the frequency of the driver. Thus the equation is saying "we can approximate the driven oscillations as having the same frequency as the driver, but with some correction ϕ".

**Figure 6:**
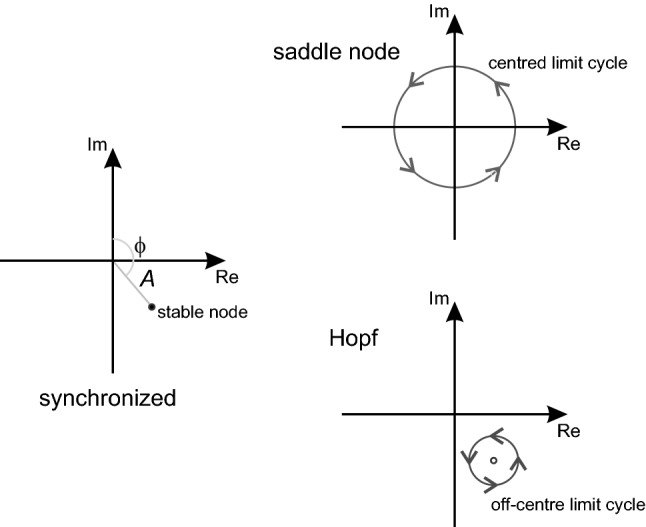
AM/FM and the dynamics of the complex truncated equation. The oscillator is a point on the complex (imaginary, real) plane. Its angular coordinates are the oscillator's envelope amplitude (*A*, radius) and its phase difference from the coupled/driving oscillator (ϕ, angle). These quantities correspond to modulation of amplitude (AM) and frequency (FM), respectively. During synchrony there is a stable node equilibrium and so both *A* and ϕ are fixed: the fixed ϕ is the definition of synchrony. Desynchronisation can occur via a supercritical Andronov–Hopf or saddle-node bifurcation. In the Hopf the stable node becomes unstable and a limit cycle grows outward from it. As the limit cycle is off the origin, both *A* and ϕ modulate periodically. In the saddle-node the stable node collides with a saddle (not shown), creating a limit cycle that is centred on/near the origin. Therefore whilst ϕ increases linearly, *A* remains near constant. Figure based on Ref.^8^ (Figs. 1, 3) and Ref.^44^ (Fig. 2). See Ref.^8^ for a more detailed explanation.

The utility of (2) is that there is a general method (called averaging) which can be used to find the differential equations for *A* and ϕ^40^. So instead of directly solving the *particular* differential equation for a *particular* oscillator, which will involve methods *particular* to that equation, one derives from that particular equation, by the *general* method of averaging, the differential equations for *A* and ϕ. These equations (for *A* and ϕ) are called the truncated equations. "Truncated" is used in the specific mathematical sense of ignoring the higher terms in an infinite series, which translated loosely means the equations for *A* and ϕ are approximations. A secondary advantage of the truncated equations is that they can be expressed as a single complex equation and this makes them even simpler to derive, solve and analyse^4^. In the complex truncated equation, *A* and ϕ are polar coordinates (radius and angle, respectively) of the oscillator on the complex plane (Fig. 6).

A number of authors have derived truncated equations for various oscillators (e.g. Equation 9b in Ref.^41^; Eq. 3 in Ref.^19^; Eq. 3.21 in Ref.^9^). These are often very similar in appearance, suggesting some underlying universality. Indeed there is a universal complex truncated equation: it is called the Stuart–Landau equation or, in its spatially-extended (partial differential) form, the Ginzburg–Landau equation^3,8,42^. The Stuart–Landau applies to any driven nonlinear oscillator. In terms of this equation, the reasoning for modulation during the supercritical Andronov–Hopf bifurcation is summarised in Fig. 6 (see Ref.^8^ for details). If the Stuart/Ginzburg–Landau is the unifying equation for synchronization then it must ultimately explain the common occurrence of periodic pulling, and the Lashinsky spectrum in particular, in so many diverse physical (and now biological) phenomena. Both AM and FM "emerge" from the dynamics expressed in the Stuart–Landau equations.

Thus the explanation for AM/FM is rather empty from a biological perspective: there is no specific biological factor to point to as a causal factor for AM and FM in the small intestine. Like so often in physics, the mathematical theory comes before we can give a mechanistic interpretation to that math in terms of concepts we already understand. In this regard the work of Hutcheon and Yarom might show the way^43^. Building on the work of earlier pioneers like Hodgkin, Huxley and Fitzhugh, they have built a theory that bridges the classical^5^ and nonlinear-dynamical^40^ theory of oscillators with the biological facts of plasma membranes and ion channels. Their ideas may be a way of translating the dynamics of the truncated equations into biological language. In particular their treatment of damping in terms of amplifying and resonating currents may be applicable to damping regimes and bifurcation across the Arnold tongue^8,44^. Another useful perspective is provided by Chua's theory of memristors, which correspond to ion channels in the biological context. Memristors can be characterised in terms of their admittance response to a stimulus current, an effect predicted from the analysis of their equilibrium as near the edge-of-chaos^45–48^.

As for the biological significance of these results, if that is taken to mean function, then there is none. In many communication devices AM/FM is specifically engineered as a means of reliably encoding information^21^. In the small intestine the ultimate goal is to push food in (mostly) one direction, toward the anus. Nature has evolved a coupled oscillator system with frequency gradient to do this: such a distributed network, resistant to local damage^49^, has a definite selective advantage^50^. AM/FM, along with frequency steps and dislocations follow from this natural selection as necessary 'side-effects' or epiphenomena with no selective advantage in and of themselves^26,51^.

AM and FM in the small intestine are further evidence that: (i) the ICC-MP of the small intestine are a network of coupled oscillators; (ii) slow/contraction waves are phase waves; and (iii) many motor patterns can be understood not just from the viewpoint of biological specifics but as the outcome of very general and simple laws.

## Methods

### Experiments

All procedures were approved and carried out in accordance with regulations of the Animal Research Ethics Board (approval no. AUP 14-12-49) of McMaster University, following the guidelines and policy statements established by the Canadian Council on Animal Care and legislation as presented in the Animals for Research Act, Ontario (1980) and administered by the Ontario Ministry of Agriculture and Food. Female CD-1 mice of at least 9 weeks old and of various strains (see below) were obtained from Charles River Laboratories (Wilmington, Massachusetts) or Jackson Laboratories (Bar Harbor, Maine) and fed ad libitum on standard chow.

### Diameter mapping

This is a standard technique in gastrointestinal motility research^52^. A length of intestine, colon or stomach is removed from the animal and placed in an 'organ bath'—a tray filled with saline solution that is kept at body temperature and oxygenated. The organ can be kept alive and functioning for a period of hours under these conditions. Video recordings are taken of the organ against a contrasting (typically black) background. The contrast allows a computer algorithm to measure the apparent width (“diameter”) of the organ at all points all its length, by simple pixel-intensity thresholding. From this data the algorithm constructs a 'diameter map' (DMap; Fig. 2). This is an image, with time running horizontally and distance along the intestine running vertically from proximal (top) to distal (bottom). The grey-scale intensity of the Dmap is the width of the intestine, from black (contracted) to white (relaxed).

### Intestine preparation

Small intestines of 14-week-old CD1 mice were prepared in chilled and oxygenated Krebs solution containing (mM) 120 NaCl, 3 KCl, 15.5 NaHCO_3_, 1.2 NaH_2_PO_4_, 0.1 citric acid, 0.1 aspartic acid, and 1.2 MgCl_2_. The entire mesentery was cut away, and a “cannula head” was inserted into the lumen at the gastroduodenal junction. The cannula head consisted of a 12-mm-long metal tube (fashioned from a 16-gauge needle) terminated by a plastic collar. The intestine was slipped over the collar and tied just behind it to prevent the intestine from slipping off the cannula head. The intestine was cut to a 25- to 30-cm length distal from the gastroduodenal junction. Typically, this would remove 5–10 cm of terminal ileum. The cannula head was connected to a reservoir of saline (135 mM NaCl–5 mM KCl), and the intestinal contents were flushed out. It was essential to keep the level of the reservoir at 10 cm above the intestine. Exertion of larger pressures prevented strong circular contractions from developing during the experiment. After the contents were flushed out, the ileal end of the intestine was also connected to a cannula head.

### Organ bath

The organ bath consisted of a plastic wallpaper tray (54–12 cm; Fig. 1B). A manifold made of metal gutter covering was attached by Velcro to the bottom of the tray. This manifold held a U-bend of 9-mm-bore Tygon tubing connected to a water circulator for heating the bath. Parallel to this, a length of PE-190 tubing, perforated and closed at one end, was installed for oxygenation. The bath was filled with 1.5 L of Krebs solution containing (mM) 120 NaCl, 5.9 KCl, 15.5 NaHCO_3_, 1.2 NaH_2_PO_4_, 0.1 citric acid, 0.1 aspartic acid, 2.5 CaCl_2_, 1.2 MgCl_2_, and 6 glucose. The bath solution was circulated by a brushless submersible micro pump (Docooler, Shenzhen, Guangdong, China). A plastic ‘T’ junction adaptor, perforated with a line of holes along the top edge and stoppered at the ends of the top edge, was attached to the outflow. This design was compact and gave a strong, approximately laminar flow. All experiments were carried out at 36 °C and in the presence of 0.5 mM lidocaine.

A “kit-kat,” a 58 × 5 cm plastic bar with three lengthwise square grooves or lanes, 1 cm wide and deep, was placed inside the U-bend. Each lane of the kit-kat held a single intestine. The intestine was held in place by insertion of its cannula heads (one on either end) into a cannula receiver, which was squeezed to fit into the lane. A Peri-Star Pro pump (World Precision Instruments, Sarasota, FL, USA) was used to pump oxygenated Krebs solution into the gastro-duodenal (proximal) end of the intestine at a rate of ~ 0.8 ml min^−1^. The inflow was warmed by passing the tube first through the bath. Outflow from the ileal (distal) end of the intestine was through Intramedic PE205 tubing (Becton Dickinson, Franklin Lakes, NJ, USA) passed through a hole in a rubber window at the end of the bath and into a beaker.

Two brackets, one at either end of the bath, held two lengths of clear plastic that partitioned the solution surface at either side of the kit-kat. This prevented passage of surface bubbles (from the U-bend oxygenation tube) over the field of the kit-kat. LED light wands at each end of the bath were used for shallow angle lighting. Illumination from above gave reflections and illumination from the sides cast shadows in the lanes of the kit-kat, interfering with spatiotemporal mapping.

Miniature CCD board cameras (1/3 = SONY Super HAD CCD, 700 TVL, and SONY Effio-E DSP), with 50-mm focal length lens (F2.0, AOV 9°), were purchased from Security Camera 2000 (Hong Kong). A row of 10 cameras were mounted 65 cm above the organ bath by means of a horizontal L-bar supported by two clamp stands. Camera signals were recorded by a 16-channel digital video recorder at 30 frames/s (model K4116HMF, Vonnic, Markham, ON, Canada).

### Analysis

Dmaps were calculated by an automated thresholding algorithm, written as a 'plugin' for ImageJ (National Institutes of Health, Bethesda, MD). The plugin (DMapLE) can be downloaded from the website of S.P. (www.scepticalphysiologist.com/code/code.html). All DMaps were bandpass filtered along the temporal axis (0.1 and 5.0 Hz cut-offs) to remove slow variations in diameter and variations across space. The analytic signal and Fourier transform were calculated with the Scipy package for Python (scipy.signal.hilbert and scipy.fftpack.fft, respectively). Instantaneous phase was calculated as the angle of the analytic signal. This was then used to calculate the difference between the instantaneous phase (ϕ) of two signals.

### Model

The model consisted of a chain of 200 coupled Van der Pol (VdP) oscillators,

3 $$\begin{gathered} \left[ {\begin{array}{*{20}c} {\dot{v}} \\ {\dot{w}} \\ \end{array} } \right]_{i} = \left[ {\begin{array}{*{20}c} {\mu (w + v - \tfrac{1}{3}v^{3} ) + k_{ij} \sum\nolimits_{j}^{{}} {(v_{j} - v)} } \\ { - \omega_{0}^{2} v/\mu } \\ \end{array} } \right]_{i} \\ i = 1 \ldots 200 \\ j = i - 1,i + 1, \\ \end{gathered}$$

where *i* indicates the ODE system of the *i*th oscillator in the chain; *j* is the index of its neighbours (*i*—1 and *i* + 1); *v* is the 'membrane potential' variable; *w* is the recovery variable; μ is the damping or timescale-separation (set to 9); *k*_*ij*_ is the coupling strength between the *i*th oscillator and its *j*th oscillator neighbour (see below); ω_0_ is the natural frequency of the oscillator. ω_0_ decreased linearly from 0.9 Hz at the proximal end to 0.75 Hz at the distal end (frequencies were converted to radians/s). This approximates the measured gradient in the small intestine.

In previous papers we showed that frequency steps likely arise at points of low coupling between ICC^25,26^. To model this we varied coupling strength (*k*) randomly along the chain^25^. *k* was symmetrical (*k*_ij_ = *k*_ji_). *k* was the product of a gap junction conductance (*g*_junc_ = 5) and gap junction density (*d*_junc_). *d*_junc_ had a reversed Lévy distribution restricted between 0 and 1,

4 $$\begin{gathered} k_{ij} = k_{ji} = g_{junc} d_{junc} \hfill \\ d_{junc} = 1 - X, \hfill \\ \end{gathered}$$

where *X* was a random variate from the Lévy stable distribution^53^ with the characteristic function,

5 $$\varphi \left( t \right) = \exp \left[ { - \sigma^{\alpha } |t|^{\alpha } \left( {1 - i\beta {\text{sgn}} (t)tan(\pi \alpha /2)} \right) + it\mu } \right]$$

with characteristic exponent α = 0.6, skewness β = 0.8, scale σ = 0.02 and location μ = 0.04. Random variates were generated with the Matlab code of Mark Veillette^54^ until a variate was in the range [0, 1).

The model was solved numerically in MATLAB using the ode45 Runge–Kutta method and model "diameter maps" (Fig. 5B) show the value of the *v* variable.

## Data Availability

All data and code is available upon request from the corresponding author. The diameter mapping software (DMapLE) is also available at https://scepticalphysiologist.com/code/code.html.
